# Rare KCND3 Loss-of-Function Mutation Associated With the SCA19/22

**DOI:** 10.3389/fnmol.2022.919199

**Published:** 2022-06-23

**Authors:** Mengjie Li, Fen Liu, Xiaoyan Hao, Yu Fan, Jiadi Li, Zhengwei Hu, Jingjing Shi, Liyuan Fan, Shuo Zhang, Dongrui Ma, Mengnan Guo, Yuming Xu, Changhe Shi

**Affiliations:** ^1^Department of Neurology, The First Affiliated Hospital of Zhengzhou University, Zhengzhou University, Zhengzhou, China; ^2^Academy of Medical Sciences of Zhengzhou University, Zhengzhou, China; ^3^Department of Cell Biology and Medical Genetics, Basic Medical College of Zhengzhou University, Zhengzhou, China; ^4^Henan Key Laboratory of Cerebrovascular Diseases, The First Affiliated Hospital of Zhengzhou University, Zhengzhou University, Zhengzhou, China; ^5^Institute of Neuroscience, Zhengzhou University, Zhengzhou, China; ^6^The Henan Medical Key Laboratory of Hereditary Neurodegenerative Diseases, The First Affiliated Hospital of Zhengzhou University, Zhengzhou University, Zhengzhou, China; ^7^The Key Laboratory of Cerebrovascular Diseases Prevention and Treatment, The First Affiliated Hospital of Zhengzhou University, Zhengzhou University, Zhengzhou, China

**Keywords:** *KCND3* mutation, SCA19/22, iPS, neuron, transcriptome (RNA-seq), endoplasmic reticulum stress, PERK-ATF4-CHOP pathway

## Abstract

Spinocerebellar ataxia 19/22 (SCA19/22) is a rare neurodegenerative disorder caused by mutations of the *KCND3* gene, which encodes the Kv4. 3 protein. Currently, only 22 *KCND3* single-nucleotide mutation sites of SCA19/22 have been reported worldwide, and detailed pathogenesis remains unclear. In this study, Sanger sequencing was used to screen 115 probands of cerebellar ataxia families in 67 patients with sporadic cerebellar ataxia and 200 healthy people to identify *KCND3* mutations. Mutant gene products showed pathogenicity damage, and the polarity was changed. Next, we established induced pluripotent stem cells (iPSCs) derived from SCA19/22 patients. Using a transcriptome sequencing technique, we found that protein processing in the endoplasmic reticulum was significantly enriched in SCA19/22-iPS-derived neurons and was closely related to endoplasmic reticulum stress (ERS) and apoptosis. In addition, Western blotting of the SCA19/22-iPS-derived neurons showed a reduction in Kv4.3; but, activation of transcription factor 4 (ATF4) and C/EBP homologous protein was increased. Therefore, the c.1130 C>T (p.T377M) mutation of the *KCND3* gene may mediate misfold and aggregation of Kv4.3, which activates the ERS and further induces neuron apoptosis involved in SCA19/22.

## Introduction

Spinocerebellar ataxia (SCA) is a group of hereditary neurodegenerative disorders characterized by progressive cerebellar ataxia and variable pyramidal, extrapyramidal, cerebral, or spinal cord symptoms in all ages (Durr, 2010), including in people with Parkinson's syndrome and epileptic symptoms. SCA has been divided into more than 40 subtypes (Soong and Morrison, 2018), and the global incidence rate is about 0–5/100,000. The specific mechanism of SCA is not yet clear. At present, the clinical treatment is mainly symptomatic support, and there is no effective treatment plan to prevent or slow the progress of SCA.

**Figure 1:**
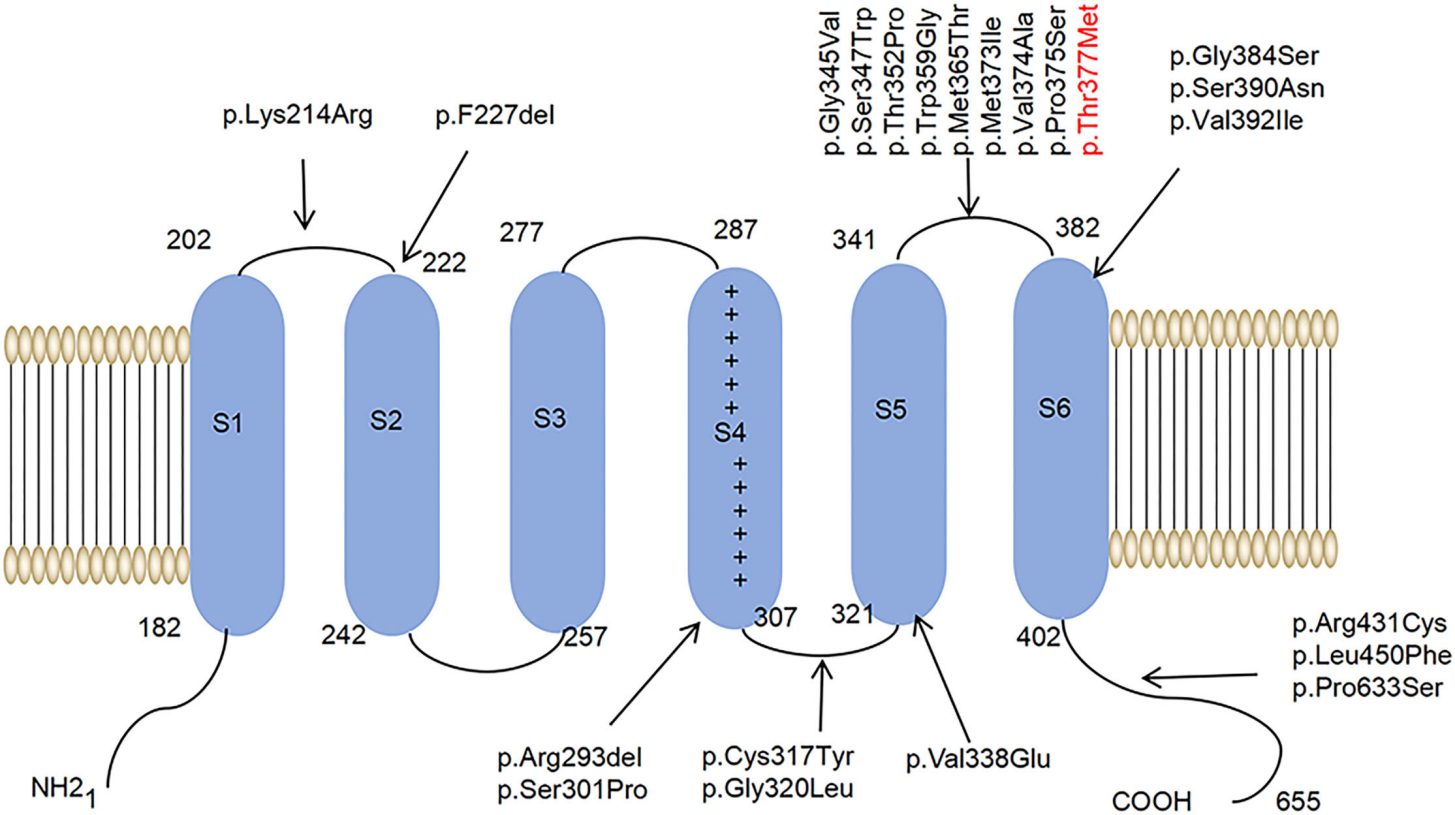
A schematic diagram of the molecular structure of Kv4.3. A Kv4.3 subunit containing six transmembrane segments (S1–S6). The arrows indicate that 22 variants have been reported worldwide.

Among the ion channels, potassium channels have the most subtypes and the most complex functions and are the focus of much clinical and scientific research. For example, research has been conducted on voltage-gated potassium channels, such as Kv1, Kv2, Kv3, and Kv4 (Heijman et al., 2021; Ikeno et al., 2021; Kleis et al., 2022). The Kv family of potassium channels are glycosylated polypeptide complexes composed of four α-subunits and four β-auxiliaries. The α-subunit is its central functional unit; each α-subunit contains one voltage receptor, and four α-subunits are combined to form a central pore. Potassium channels are widely distributed in the body and are related to various neurodegenerative diseases (Priest et al., 2021; Sancho and Kyle, 2021). Kv4.3 is a potassium channel protein that is encoded by the *KCND3* gene, which expresses in the deep nucleus, granulosa cells, and Purkinje cells, as well as in various intermediate neurons in the cerebellum, cerebral cortex, hippocampus, and pons (Isbrandt et al., 2000; Ohya et al., 2001; Kollo et al., 2006). Studies have reported that potassium channels play a role in the postnatal migration of cerebellar neurons (Hsu et al., 2003). Kv4.3 is activated and inactivated rapidly after membrane depolarization, promoting the sub-threshold A-type potassium current that controls the repolarization and frequency of action potential, thus preventing the excitability of neurons (Serôdio et al., 1996; Niwa and Nerbonne, 2010). (See Figure 1 for a schematic diagram of the molecular structure of Kv4.3.)

It has been proved that SCA type 19/22 (SCA19/22) is associated with mutations in the *KCND3* gene located in 1p21-q21 (OMIM:607346) (Lee et al., 2012). Moreover, the variants of *KCND3* are related to sudden unexpected death syndrome (SUDS) and tardive Brugada syndrome-9 (BRGDA9) (Giudicessi et al., 2012; Duarri et al., 2015). So far, 22 single-nucleotide mutation sites of SCA19/22 have been reported worldwide. The clinical phenotypes of SCA19/22 are complex and may be related to different mutation sites.

Some research has reported that pathogenic mutations promoted the misfolding and aggregation of disease-causing proteins in SCA5 and SCA14 (Armbrust et al., 2014; Takahashi et al., 2015; Avery et al., 2017), which further activated the endoplasmic reticulum stress (ERS) pathway and eventually led to neuronal dysfunction. The endoplasmic reticulum recognizes correctly folded proteins and transports them to the Golgi apparatus. ERS is the response of cells to unfolded protein gathered in the endoplasmic reticulum and calcium ion disorder, which further activates signal pathways such as UPR, endoplasmic reticulum overload response, and neuron apoptosis (Oakes and Papa, 2015). ERS has been associated with various chronic neurodegenerative diseases and the accumulation of misfolded proteins accompanied by ERS-induced neuronal dysfunction (Sprenkle et al., 2017).

Nevertheless, the exact molecular pathophysiology of SCA19/22 is still unclear due to the scarcity of clinical samples and the relatively less research in this area. Therefore, we performed a screening targeting *KCND3* in a cohort of undiagnosed cerebellar ataxia patients. Next, we explored the detailed pathogenesis of SCA19/22 with *KCND3* loci mutation by inducing pluripotent stem cells (iPSCs) derived from patients, which may serve as drug targets for developing potential SCA19/22 therapeutics.

## Materials and Methods

### Subjects

All of the participants gave their written informed consent, and the ethics committee of the First Affiliated Hospital of Zhengzhou University approved this study.

We performed a screening targeting *KCND3* in a cohort of undiagnosed cerebellar ataxia patients, including the families of 115 probands and 67 sporadic cerebellar ataxia patients; all of the participants were outpatients in the Department of Neurology at the First Affiliated Hospital of Zhengzhou University. SCA 1, 2, 3, 6, 7, 12, and 17 and dentatorubral–pallidoluysian atrophy (DRPLA), which are common in China, were excluded in advance. Two hundred healthy Chinese volunteers were analyzed as sex- and age-matched controls. The clinical evaluations of all the participants are shown in Table 1. Peripheral blood samples were collected from all of the participants. Genomic DNA was extracted from peripheral blood with a medium quantity whole blood genomic DNA isolation kit (BioTeke Corporation, Cat. #DP2102, China). Sanger sequencing samples were sent to SUNYA Co., Ltd. (http://www.zjsyswjs.com/). All of the patients underwent a thorough neurological examination by two professional neurology specialists. The following scales were used during the clinical evaluation: the Assessment and Rating of Ataxia (SARA), the International Cooperative Ataxia Rating Scale (ICARS), the MDS-Unified Parkinson's Disease Rating Scale (MDS-UPDRS), and the Mini-Mental State Examination (MMSE). Magnetic resonance imaging (MRI) was used to observe the cerebellum structure.

**Table 1:** The clinical evaluations of all the participants.

**Category**	**Patients**	**Healthy controls**
Gender (Female/male)	81/101	92/108
Age (year)	45.27 ± 11.69	45.27 ± 11.69
Age of onset (year)	35.18 ± 10.75	
Disease duration (year)	8.39 ± 6.84	
SARA scores	18.16 ± 9.51	
ICARS	32.49 ± 13.02	

### Pathogenicity Assessment and Protein Model Prediction

Sorting intolerant from tolerant (SIFT) (Kumar et al., 2009) and PolyPhen-2 (Adzhubei et al., 2010) were used to assess the pathogenicity of mutations. The protein sequence alignment and the protein models of conservation analysis were performed using Molecular Evolutionary Genetics Analysis software and the ConSurf Server (https://consurf.tau.ac.il). We predicted the protein structure models using the AlphaFold Protein Structure Database (https://alphafold.ebi.ac.uk/). The protein model was based on the prediction, and the figures were prepared with PyMOL 2.5.2.

### Cell Culture and Neuron Differentiation

After local anesthesia, 5-mm diameter biopsy specimens were obtained from 10 cm above the lateral malleolus of the study participants. The skin tissues were cultured at 37°C in a 5% CO_2_ atmosphere with Dulbecco's modified Eagle medium (DMEM; GIBCO, Cat. #C11995500BT, USA) containing 10% fetal bovine serum (GIBCO, Cat. #10099141C, USA) and 1% penicillin and streptomycin (Solarbio, Cat. #P7630, China); the culture medium was changed every 3 days. Fibroblasts were obtained after the skin tissues had been cultured for 15 days; the fibroblasts were cultured using the same method as was used for the skin tissues. Then, we induced the fibroblasts into iPSCs with a CytoTune™-iPS 2.0 Sendai Reprogramming Kit (ThermoFisher, Cat. #A16517, USA). Next, neural progenitor cells (NPCs) were induced from the iPSCs with a STEMdiff™ SMADi Neural Induction Kit (STEM CELL, Cat. #08581, Canada). Finally, we obtained neurons from the NPCs with a BrainPhys™ hPSC Neuron Kit (STEM CELL, Cat. #05795, Canada) following the instructions provided in the product manual. The neurons were cultured at 37°C with a 5% CO_2_ atmosphere using a BrainPhys™ hPSC Neuron Kit (STEM CELL, Catalog #08581, Canada).

### Immunofluorescence

The iPSCs, NPCs, and neurons were cultured on coverslips, washed with PBS three times for 5 min each time, and then fixed with 4% paraformaldehyde (Service, Cat. #G1101, China) for 30 min. They were then washed with PBS three times for 5 min each time with 0.1% Triton X-100 (Solarbio, Cat. #T8200, China), washed again with permeabilized cells for 15 min, PBS-washed again in 5% bovine serum albumin (Solarbio, Cat. #A8010, China), and finally blocked for 30 min at room temperature. Primary antibodies were appropriately incubated overnight at 4°C. The primary antibodies included OCT4 (1:200; Cell Signaling Technology), Nanog (1:1,000; Cell Signaling Technology), TRA-1-60 (1:1,000; Cell Signaling Technology), NESTIN1 (1:1,000; Cell Signaling Technology), PAX6 (1:200; Cell Signaling Technology), GFAP (1:200, Proteintech), MAP2 (1:200, Proteintech), Calnexin (1:200, Sigma-Aldrich), and Kv4.3 (1:500, Omnimabs). Immunoreactivities were visualized with goat anti-mouse antibodies conjugated to Alexa Fluor 594 (1:200; Abcam) and goat anti-rabbit antibodies conjugated to Alexa Fluor 488 (1:200; Abcam). A Nikon laser scanning confocal microscope (ECLIPSE Ni-U) was used to acquire fluorescence images.

### Differential Gene Expression and Pathway Analysis

We used OmicShare tools to analyze the transcriptome sequencing result and obtain differentially expressed genes (DEGs). The gene screening criteria for each sample were as follows: 1) a |log2 (FC)| >1 and 2) adjusted *P* < 0.05. The microarray data are provided in Supplementary Material 1. The heatmap for the top 100 DEGs was created using R 4.11. We drew the volcano map and the heatmap of the DEGs using the OmicShare tools. Gene Ontology (GO) annotation and the Kyoto Encyclopedia of Genes and Genomes (KEGG) were used to conduct pathway enrichment analyses of the DEGs by annotation, visualization, and integrated discovery (DAVID) (selected with an enrichment significance evaluated at *p* < 0.05) (https://david.ncifcrf.gov/tools.jsp), which revealed the biological process (BP), cellular component (CC), molecular function (MF), and pathways.

### Western Blotting

Neurons were lysed at 30 days with radioimmunoprecipitation assay (RIPA) lysis (Solarbio, Cat. #R0010, China), PMSF (Solarbio, Cat. # P0100, China), and phosphatase inhibitor (Solarbio, China) in proportions of 100:1:1, respectively. A BCA Protein Assay Kit (KeyGEN, Cat. #KGP902, China) was used for protein quantification. The proteins were used with 4–20% sodium dodecyl sulfate–polyacrylamide gel for electrophoresis and were transferred into the PVDF membrane (Merck Millipore, Cat. #ISEQ00010, Germany). They were then blocked with 5% nonfat milk for 90 min and incubated overnight with the primary antibody at 4°C. The following day, they were incubated with the secondary antibody. After that, an Omni-ECL™ Enhanced Pico Light Chemiluminescence Kit (EpiZyme, Cat. #SQ101, China) was added to capture the protein with a ChemiDoc™ MP Image System (Bio-RAD, Universal Hood III). The information about the antibodies is as follows: Kv4.3 (1:200; Affinity Biosciences); activating transcription factor 4 (ATF4) (1:200; Cell Signaling Technology); and C/EBP homologous protein (Bushart et al., 2018) (1:200, Proteintech). Goat anti-mouse IgG (H+L) -HRP (1:10,000; Bioworld) and goat anti-rabbit IgG (H+L) HRP (1:10,000; Bioworld) were used for Western blotting.

### Extraction of Neuronal MRNA for Transcriptome Sequencing and RT-QPCR

The RNA was extracted with TRIzol (ThermoFisher, Cat. #15596026, USA.). The mRNA was sent to Biomarker Technologies Corporation (http://www.biomarker.com.cn/) for full-length transcriptome sequencing. We obtained the mRNA with a FastPure Cell/Tissue Total RNA Isolation Kit V2 (Vazyme, RC112-01, China). The cDNA was prepared with HiScript III RT SuperMix for qPCR (Vazyme, R323-01, China). Taq Pro Universal SYBR qPCR Master Mix (Vazyme, Q712-02, China) on a QuantStudio 5 Real-Time PCR Instrument (Applied Biosystems) was used for quantification. The 2^−Δ*ΔCt*^ method was used to determine the relative expression of each gene, with GAPDH as a reference. The primers used to amplify the target genes by RT-qPCR are as follows: KCND3 F (AAGAACAAGCGGCAGGATGA) and R (GAGGCACAGCTCTTCAGTGT), CHOP F (TCTGGCTTGGCTGACTGAGGAG) and R (TCTGACTGGAATCTGGAGAGTGAGG), ATF4 F (CCCAGCAGACTTCACATGT) and R (CCTCCCATTTCCCTCGTTTT), and GAPDH F (GTGGACCTGACCTGCCGTCT) and R(GGAGGAGTGGGTGTCGCTGT).

## Results

### Clinical Investigation of Patients With Disease-Associated *KCND3* Mutations

**Figure 2:**
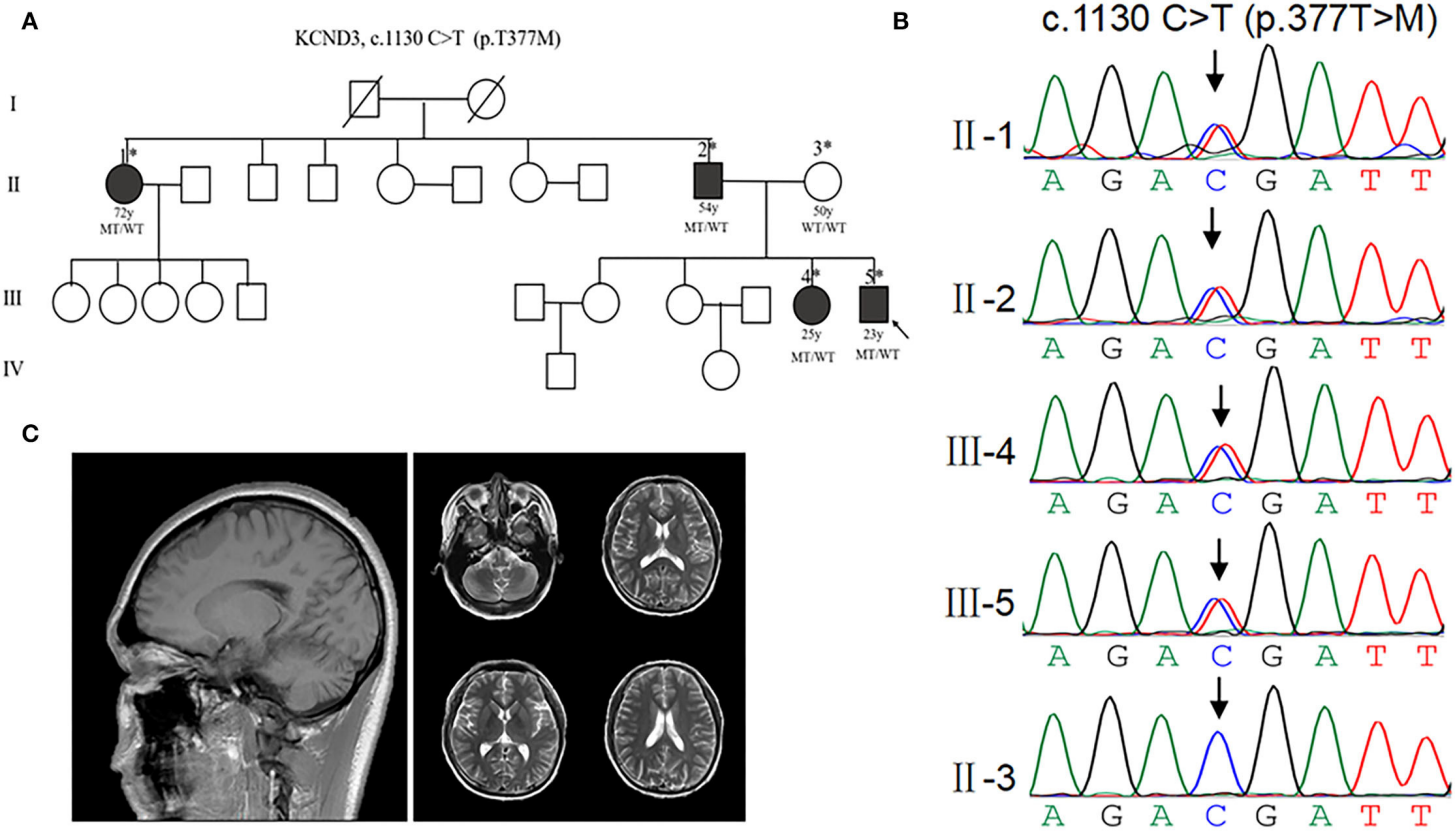
Clinical characterization of *KCND3* mutations. **(A)** Pedigree chart: there are four patients in this family. The asterisks indicate that the members have been sequenced. The arrows denote probands. The filled symbols represent symptomatic members. The open symbols indicate unaffected individuals. The circles indicate female participants. The squares indicate male participants. The diagonal lines refer to the deceased. WT/WT refers to wild-type, while MT/WT specifies heterozygous mutation. **(B)** Sequencing chromatograms. Sanger sequencing verified the c.1130 C>T (p.T377M) mutation of the *KCND3* and showed pedigree co-separation in this family. This mutation of *KCND3* was not found in the normal family members. **(C)** No cerebellar atrophy and white matter abnormalities were found in III-5's brain MRI.

To investigate the patients with *KCND3* mutation, we performed a screening targeting *KCND3* in a cohort of undiagnosed cerebellar ataxia patients, including the families of 115 probands and 67 sporadic cerebellar ataxia patients. No SCA 1, 2, 3, 6, 7, 12, and 17 or DRPLA-related mutations were detected. We identified a mutation of *KCND3*-NM_004890, c.1130 C>T (p.T377M) in one proband among all the participants. Sanger sequencing verified the screening results and showed pedigree co-separation in this family (Figures 2A,B). This mutation of *KCND3* was not found in the normal family members or in the 200 healthy controls. The proband's parents (III-4) were not closely related, and the mother was normal during pregnancy, delivery, and the perinatal period. The proband (III-4) presented with a slowly progressive head tremor since the age of 14 years. At the age of 23 years, he was treated for paroxysmal walking instability, head tremor, lead-pipe rigidity of the extremities without cerebellar atrophy, and white matter abnormalities in his MRI (Figure 2C). His sister (III-3) had been experiencing intermittent head tremors since the age of 22 years. His father had started experiencing head tremors at the age of 54 years, but the symptoms had abated after 4 years. His aunt (II-1) had experienced similar symptoms as the proband starting at the age of 52 years, and the head tremor symptom had improved after 10 years. Other members of this family had no neurological symptoms. The scale results showed that the proband scored 15/40 points on the SARA, and 10 points on the ICARS. His sister scored 13/40 points on the SARA, while his father scored 12/40 points, and his aunt scored 10 points. All of the participants scored 30/30 points on the MMSE, and no degradation of intellectual ability was found.

### Pathogenicity Assessment of *KCND3* Variants and Structural Change of Kv4.3

**Figure 3:**
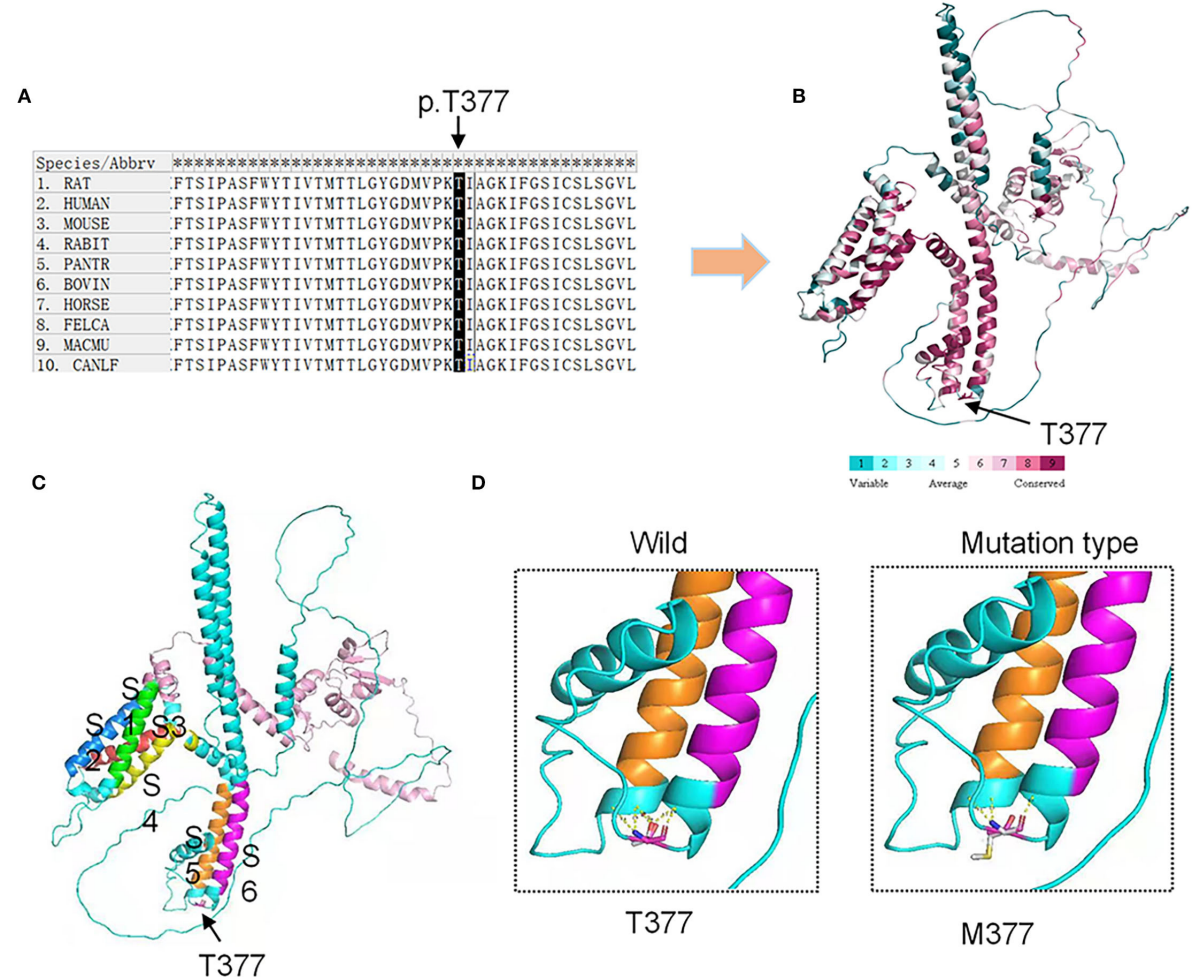
Conservation analysis and protein model prediction: **(A)** The protein sequence alignment is shown on the left. **(B)** The protein model showed that it was highly conservative. Highly conservative areas are shown in red color; the higher the score, the darker the color. **(C)** The protein structure model: A Kv4.3 subunit containing six transmembrane segments (S1–S6). The transmembrane segments are colored in green (S1), blue (S2), red (S3), yellow (S4), orange (S5), and purple (S6). **(D)** The AlphaFold Protein Structure Database's Kv4.3 structure models indicate that replacing polar threonine with nonpolar methionine causes an enlargement in the mean volume at residue 377 compared with the wild model.

We used SIFT and PolyPhen-2 to confirm the potential pathogenicity of the *KCND3* variants, and the prediction results showed that the variants of the c.1130 C>T (p.T377M) mutation of *KCND3* were damaging (Table 2). The protein sequence alignment of multiple species suggests that the variant site of the c.1130 C>T (p.T377M) mutation of *KCND3* was consistent (Figure 3A), and the conservatism analysis showed that the mutant amino acid of p.T377M was highly conservative (Figure 3B). The AlphaFold Protein Structure Database's Kv4.3 structure models indicate that replacing polar threonine with nonpolar methionine causes an enlargement in the mean volume at residue 377 compared with the wild model (Figures 3C,D).

**Table 2:** The prediction of pathogenicity at different mutation sites of KCND3.

**Transcript ID**	**Variant sequence**		**Functional prediction**	
	**Mutation position**	**Amino acid**	**SIFT**	**Polyphen2**
NM_004980.4	c.1034 G>T	p.G345V	Damage(0.045)	Benign(0.447)
	c.1013 T>C	p.V338E	Damage(0.000)	probably damaging(0.999)
	c.1130 C>T	p.T377M	Damage(0.000)	probably damaging(1)
	c.1150 G>A	p.G384S	Damage(0.004)	probably damaging(0.995)
	c.1121 T>C	p.V374A	Damage(0.002)	probably damaging(0.999)
	c.1040 C>G	p.S347W	Damage(0.004)	possibly damaging(0.952)
	c.1075 T>G	p.W359G	Damage(0.000)	probably damaging(1)
	c.950 G>A	p.C317V	Damage(0.000)	probably damaging(1)
	c.1123 C>T	p.P375S	Damage(0.002)	probably damaging(1)
	c.641 A>G	p.K214R	Tolerance(0.326)	Benign (0)
	c.1348 C>T	p.L450F	Tolerance(0.177)	Benign (0.045)
	c.1897 C>T c.950 G>A	p.P633S p.C317T	Tolerance(0.214) Damage(0.002)	Benign (0.002) possibly damaging (0.912)
	c.1094 T>C c.1291C>T	p.M365T p.R431C	Damage(0.028) Damage(0.000)	possibly damaging(0.944) probably damaging (1.000)
	c.1174 G>A	p.V392I	Tolerance(0.070)	probably damaging(0.999)
	c.1034 G>T	p.S301P	Damage(0.003)	probably damaging(0.999)
	c.1054 A>C	p.T352P	Damage(0.002)	probably damaging(0.997)
	c.1119 G>A	p.M373I	Tolerance(0.165)	Benign(0.333)
	c.1169 G>A	p.S390N	Damage(0.003)	probably damaging(0.998)
	c.679-681 del TTC	p.F227del	NA	NA
	c.877-885 dup CGCGTCTTC	p.A293del	NA	NA
NM_172198.3	c.1040 C>G	p.S347W	Damage(0.004)	possibly damaging(0.952)
	c.1075 T>G	p.W359G	Damage(0.000)	probably damaging(1)

### Cell Identification and Enrichment Analysis of Neuron Transcriptome Sequencing

**Figure 4:**
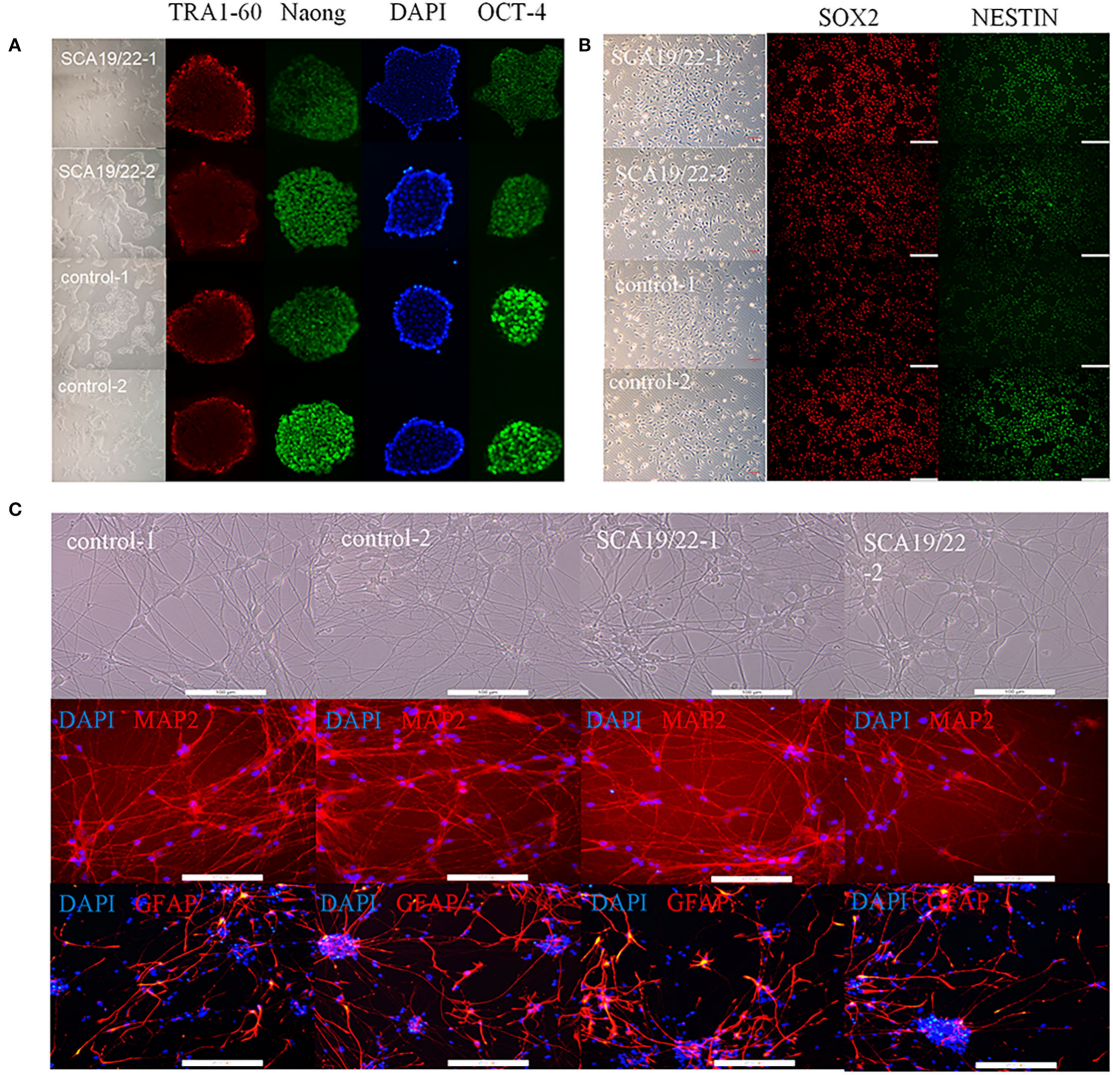
Characterization of representative SCA19/22-iPSCs and control iPSCs. Immunostaining shows **(A)** pluripotency-associated markers in representative iPSC colonies; **(B)** NPC-expressed markers such as NESTIN1 and SOX2; **(C)** neuron-expressed markers such as GFAP and MAP2. Scale bar: 100 μm for panels. iPSCs: induced pluripotent stem cells; NPC: neural progenitor cell.

**Figure 5:**
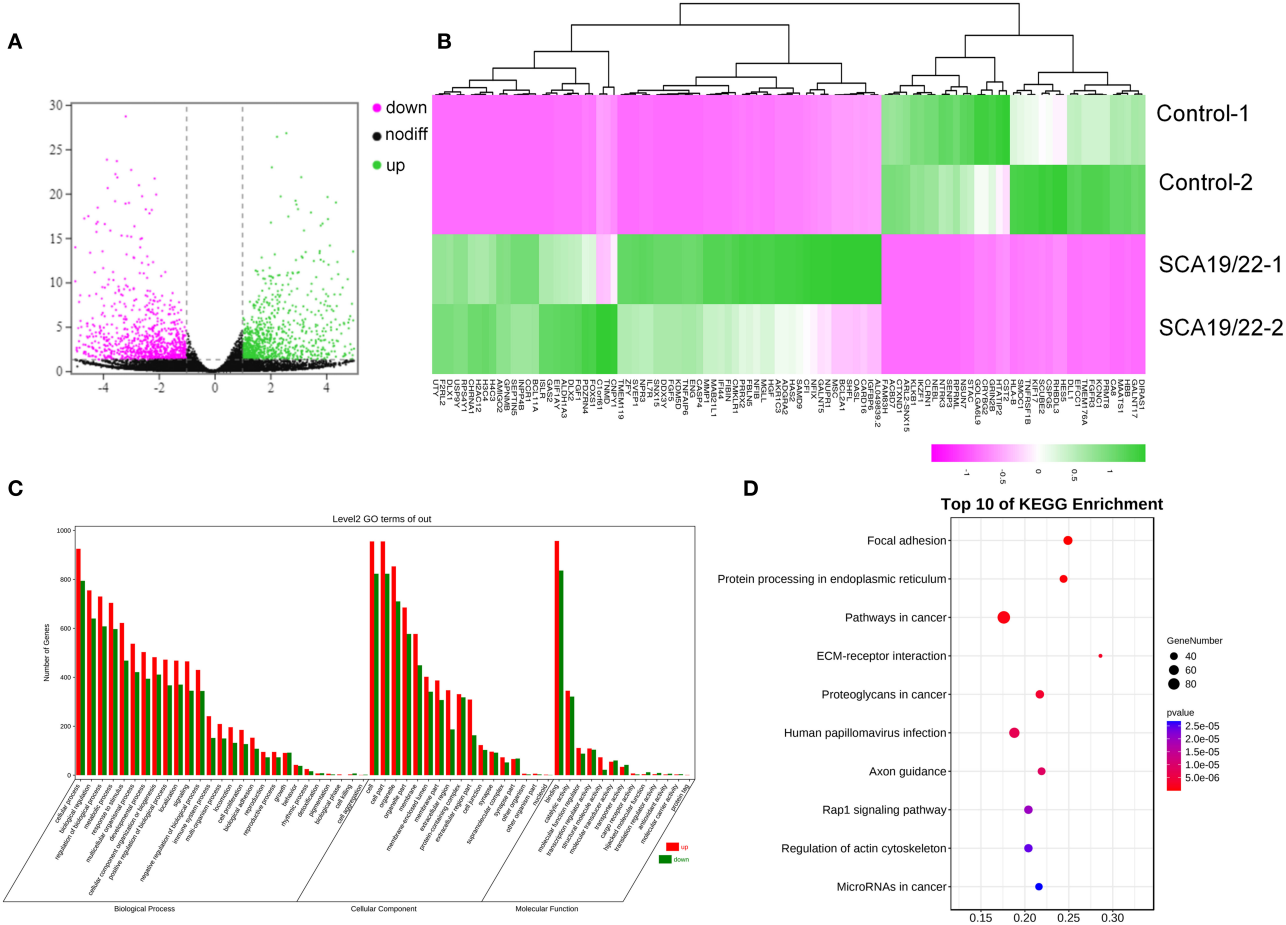
A visualization of the transcriptome data analysis. The volcano map **(A)** and heatmap **(B)** of the gene expression. The red gene is upregulated and the green gene is downregulated. |log2 (FC)| >1 is considered to be significant. Significant enrichment of GO (BP, CC, and MF) **(C)**; the KEGG pathway showed protein processing in the endoplasmic reticulum, and focal adhesion was significantly enriched **(D)**.

To further understand the pathogenesis of this mutation, we established a human SCA19/22 neuron disease model in which the *in vitro* neuron differentiation of iPSCs derived from fibroblasts from the proband and his father. Two healthy neuron lines were used as matched controls. The SCA19/22 patients (*n* = 2) and the healthy participants (*n* = 2) were divided into two groups. First, we obtained iPSCs from the fibroblasts (Figure 4A; Liu et al., 2021). Second, NPCs were induced from the iPSCs (Figure 4B). Finally, the NPCs were differentiated into neurons (Figure 4C). All the tested iPSC lines expressed pluripotency-associated markers, such as OCT4, Nanog, and TRA-1-60 (Figure 4A), while the NSC lines expressed two markers, namely, NESTIN1 and PAX6 (Figure 4B), and four neuron lines expressed GFAP and MAP2 (Figure 4C), which suggested that the skin tissues were successfully induced into neurons. Colocalization with the endoplasmic reticulum marker calnexin (in green) revealed that mutant Kv4.3 protein (in red) was retained in the NPCs, as was shown by the merged picture (in yellow). The results of fluorescence intensity analysis showed that more Kv4.3 was trapped in the NPCs in the disease group (Supplementary Figure 1). Then, the mRNA of neurons was sent to Biomarker Technologies Corporation for full-length transcriptome sequencing. Unknown genes in the RNA-sequence data were removed in advance. The RNA-sequence data of the two groups were analyzed using the DESeq2 method to obtain 1,958 DEGs (|Log2FC|>1, *p* < 0.05), including 929 downregulated and 1,029 upregulated genes. *KCND3* was not significantly changed. This result is shown in the heatmap and volcano map of the DEGs created using the OmicShare tools (Figures 5A,B). The DAVID 6.8 online tool was used to perform functional and pathway enrichment analyses with all DEGs. The GO and KEGG items, including BP, CC, MF, and KEGG pathways, were significantly enriched (Figures 5C,D). The enriched KEGG category contained three pathways, namely, focal adhesion, protein processing in the endoplasmic reticulum, and axon guidance (Figure 5D). These pathways were closely related to ERS, apoptosis, and neuronal growth.

### The Changes in Kv4.3, ATF4, and CHOP in Neurons

**Figure 6:**
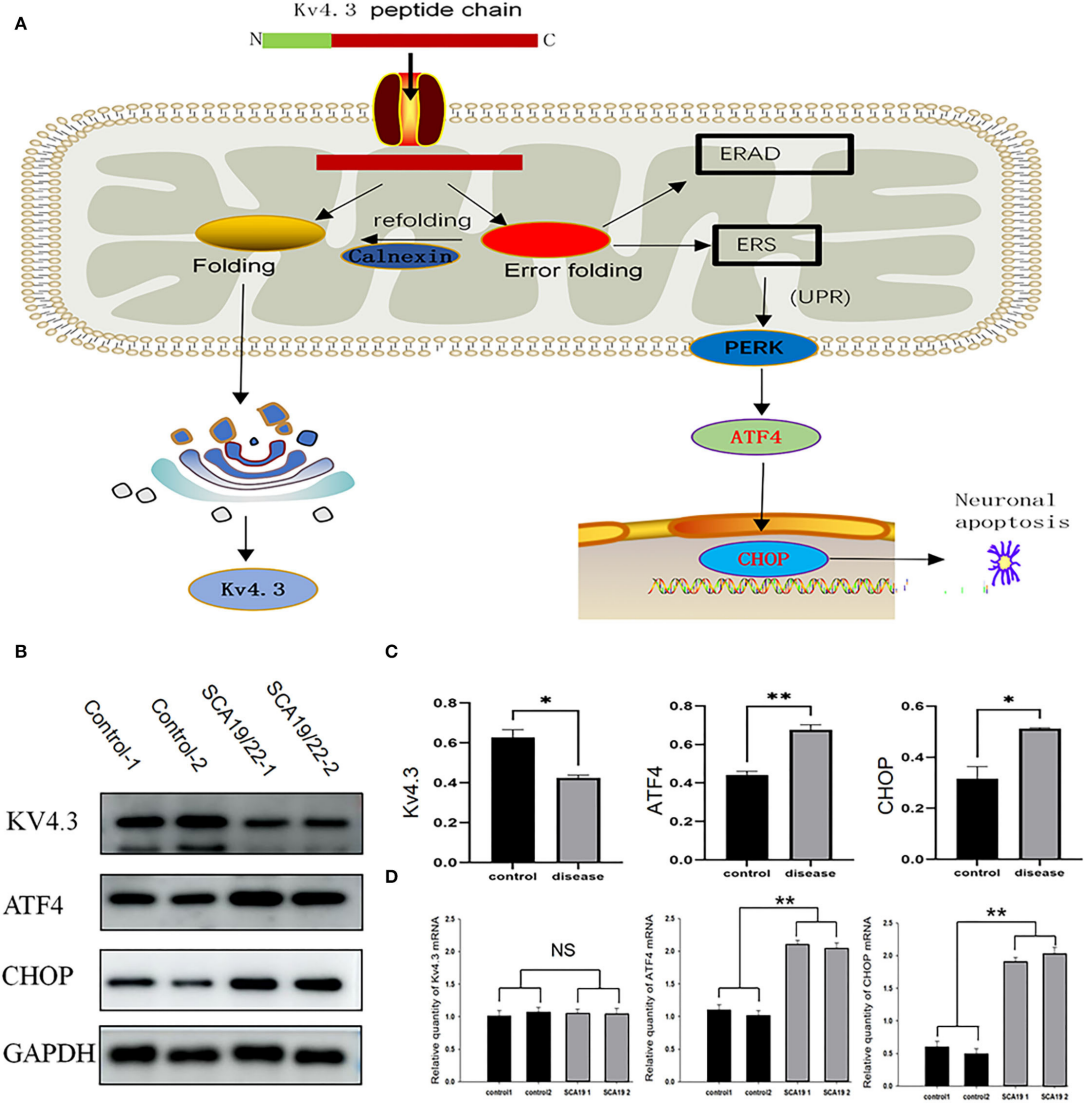
**(A)** The mutant Kv4.3 instability increased and remained in the endoplasmic reticulum, which may activate the endoplasmic reticulum-associated degradation (ERAD) pathway and cause ERS. Increased expression of ATF4 and CHOP can eventually lead to neuronal death. **(B)** Total proteins derived from neurons were subject to immunoblotting analyses with the indicated antibodies. GAPDH expression was the loading control. **(C)** The Kv4.3, ATF4, and CHOP levels were quantified and normalized to the GAPDH. Western blotting showed that the c.1130 C>T (p.T377M) mutation of *KCND3* led to a decrease in Kv4.3 protein compared with the healthy controls (*p* < 0.05), while there was a significant increase in ATF4 and CHOP (*p* < 0.05). **(D)** The mRNA levels of Kv4.3, ATF4, and CHOP were quantified and normalized to the GAPDH. RT-qPCR showed that the mRNA level of mutated *KCND3* was not significantly different from that of the healthy controls. However, the mRNA levels of ATF4 and CHOP were significantly increased (*p* < 0.05). Data are expressed as the mean ± SD; The significance was calculated using Student's *t*-test (*p < 0.05; **p < 0.01).

We selected ATF4 and CHOP to verify the function of ERS in the pathogenesis of SCA19/22. Western blotting showed that the c.1130 C>T (p.T377M) mutation of *KCND3* led to a decrease in Kv4.3 protein compared with the healthy controls (*p* < 0.05), while there was a significant increase in ATF4 and CHOP (*p* < 0.05; Figures 6B,C). The original exposure picture is provided in Supplementary Material 3. RT-qPCR showed that the mRNA level of mutated *KCND3* was not significantly different from that of the healthy controls. However, the mRNA levels of ATF4 and CHOP were significantly increased (*p* < 0.05; Figure 6D).

## Discussion

**Figure 7:**
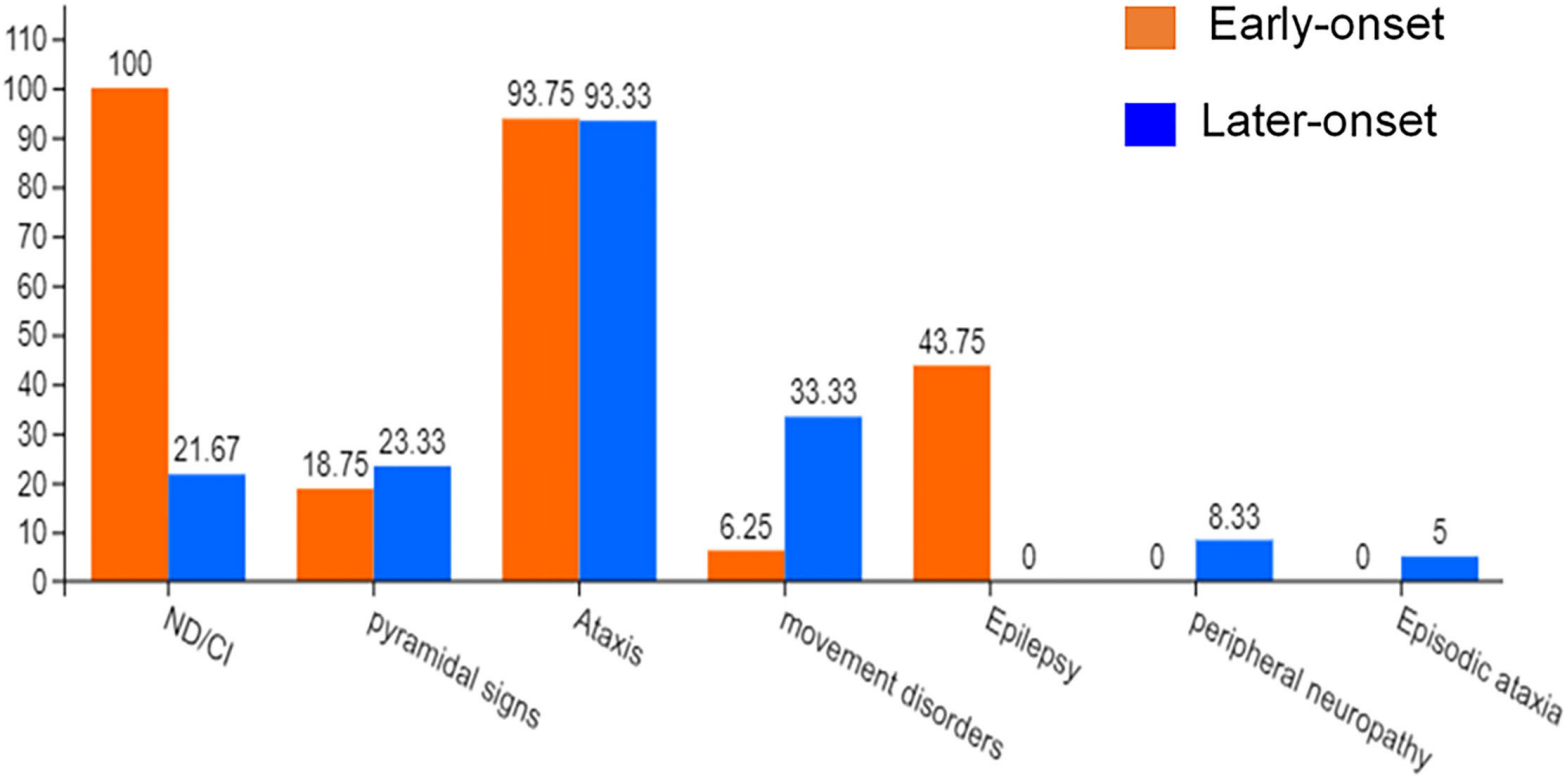
The percentage of symptoms in the early-onset and late-onset cohort. ND, Neurodevelopmental disorders; CI, Cognitive impairment.

Previous case reports of *KCND3* variations included 76 patients, including 16 early-onset patients and 60 late-onset patients (Pollini et al., 2020; Paucar et al., 2021; Zanni et al., 2021; Ha et al., 2022). The clinical data of 76 *KCND3* mutation carriers are shown in Supplementary Material 4. The percentage of symptoms in the early and late cohorts are shown in Figure 7. The progress of late-onset SCA19/22 is slow, and during the follow-up of some patients, the SARA score increased by an average of 0–2 points per year. However, there is a significant difference in the severity and progression rate of the disease in early-onset SCA19/22 (Smets et al., 2015; Huin et al., 2017). Among these patients, cerebellar atrophy (35/35) was the primary manifestation of brain MRI in late-onset patients, and the brain MRI of the early-onset patients was found normal (5/6).

Purkinje neuron loss occurred in most SCA subtypes (Chen et al., 2012; Seidel et al., 2012; Adachi et al., 2015; Koeppen, 2018). The main mechanisms of Purkinje neuron loss include proteotoxicity (Alves et al., 2014; Ashkenazi et al., 2017), RNA toxicity (Ishiguro et al., 2017; Zhang and Ashizawa, 2017), and channel dysfunction (Coutelier et al., 2017; Dell'Orco et al., 2017). Proteotoxicity is mainly manifested in the formation and toxicity of abnormal protein aggregates in abnormal cells (Ashkenazi et al., 2017), or the structure of the mutant protein changes, which reduces the synaptic stability of Purkinje neurons (Avery et al., 2017). Channel dysfunction is characterized by the dysfunction of the electrical activity of Purkinje neurons (Duarri et al., 2012). Some studies have revealed that the related pathological changes of *KCND3* variants mainly include impaired protein trafficking, increased protein degradation, and reduced outward K^+^ current (Duarri et al., 2012; Hsiao et al., 2019). The mutant Kv4.3 instability increased and remained in the endoplasmic reticulum, which may activate the endoplasmic reticulum-associated degradation (ERAD) pathway and cause ERS.

In our study, the c.1130 C>T (p.T377M) mutation of the *KCND3* gene was reported in the Chinese mainland family for the first time. To further explore the pathogenesis of SCA19/22, we obtained SCA19/22-iPSC-derived neurons as a patient-derived neuron model might explain the initial characteristics of SCA19/22 better than the mouse model and other disease models of non-human origin and might provide a more detailed understanding of the pathogenesis of SCA19/22 caused by the *KCND3* c.1130 C>T (p.T377M) mutation. Moreover, we revealed for the first time the role of ERS in the pathogenesis of SCA19/22 by the patient-derived iPSCs combined with transcriptome sequencing.

We summarized 22 *KCND3* single-nucleotide mutation sites of SCA19/22 worldwide (Table 2); the clinical overview is listed in Table 3. No cerebellar atrophy or white matter abnormalities in MRI of the proband was observed in this study. The MMSE evaluation showed no degradation of intellectual ability. Although no effective treatment had been applied, the head tremor of II-1 and II-2 had been relieved compared with the onset of the disease, which was different from previous reports (Lee et al., 2012; Paucar et al., 2018; Hsiao et al., 2019). It may be that different mutation loci lead to different changes in the function and structure of the same protein. The progress of the disease is related to the mutation type.

**Table 3:** Clinical data of *KCND3* mutation carriers.

**Mutations**	**Country**	**Patients**	**Age of onset (years)**	**SARA**	**MRI**	**Clinical symptoms**
p.T377M	China	4	14–54	10–15	Normal	Episodic ataxia, Head tremor, Dystonia, Paroxysmal walking instability,
	Sweden	7	child-18	5–24	Vermian cerebellar atrophy;White matter lesions	Somatic ataxia, Cognitive impairment
	Japan	4	10–40	29	Mild cerebellar atrophy	Episodic ataxia, Dysarthria,Nystagmus, Tendon hyperreflexia
p.G320L	China	1	1	7	Normal	Episodic ataxia, Mental retardation, Dystonia, Tendon hyperreflexia
F227del	China	14	15–46	9	Mild cerebellar atrophy	Cerebellar ataxia
	France	8	24–51	23	Mild cerebellar atrophy	Ataxia, Palsy of upper eyelid muscle, Diplopia, Sensory abnormality, Urinary incontinence
p.C317V	China	1	15	40	Cerebral atrophy; Hemispheric cerebellar atrophy;Vermian cerebellar atrophy	Ataxia, Dystonia
p.P375S	China	1	36	10–15	Cerebral atrophy; Hemispheric cerebellar atrophy;Vermian cerebellar atrophy	Ataxia, Cognitive impairment
p.S301P	Italy	1	3	27	Vermian cerebellar atrophy	Episodic ataxia, Dysphonia, Mental retardation, Neurodevelopmental disorders,Epilepsy, Parkinson's syndrome
p.T352P	Netherlands	13	1–45	23	Cerebral atrophy; Hemispheric cerebellar atrophy;Vermian cerebellar atrophy	Episodic ataxia, Cognitive impairment, Myoclonus, Head tremor, Nystagmus
p.M373I	Netherlands	2	44–64	12	Mild cerebellar atrophy	Episodic ataxia
p.S390N	Netherlands	3	30–35	25	Vermian cerebellar atrophy	Spastic ataxia, Dysarthria, Nystagmus, cognitive impairment, Hearing impairment,
p.G345V	Germany	4	35–50	10	Vermian cerebellar atrophy	Episodic ataxia, Dysarthria
	Japan	4	45–55	11	Vermian cerebellar atrophy	Ataxia, Dysarthria
p.V338E	Japan	3	51–90	1	NA	Ataxia, Dysarthria, Cognitive impairment
R293_F295_dup	Belgium	1	3	31	NA	Ataxia, Mental retardation, Dysphagia, Dysarthria, Nystagmus, Epilepsy
p.L450P	France	1	39	19	NA	Episodic ataxia, Pyramidal sign, Brugada symptoms
p.K214R	France	1	<30		Vermian cerebellar atrophy	Intermittent gait disorder, Vertigo, Sensory abnormality, Hoffmann positive, Nystagmus

Although the pathogenic genes are different in each case, the SCA subtypes have some common physiological characteristics, such as protein aggregation, dysregulated autophagy, ion channel defects, mitochondrial defects, transcriptional dysregulation, and neuronal cell death (Durr, 2010; Klockgether et al., 2019). The results of neuronal transcriptome sequencing revealed that the differential expression of genes was significantly enriched in focal adhesion, protein processing in the endoplasmic reticulum, and axon guidance, which were mainly related to ERS, apoptosis, and neuronal growth (Cance and Golubovskaya, 2008; Hetz and Saxena, 2017; Howard et al., 2019). In the SCA19/22-derived neurons, the Kv4.3 protein was decreased, which was consistent with previous research results (Zanni et al., 2021). However, there was no significant difference in the mRNA level of *KCND3*. The Kv4.3 conservation analysis and protein prediction model indicated that the *KCND3* c.1130 C>T (p.T377M) mutation led to a polar change in the mutant amino acid site. We also observed that the mRNA and protein levels of CHOP and ATF4 were significantly upregulated, suggesting that the UPR and PERK-ATF4-CHOP pathways were activated. ATF4 and CHOP played a crucial role in activating the PERK-ATF4-CHOP pathway and eventually led to Bcl2-mediated neuron apoptosis (Han et al., 2013; Aimé et al., 2020; Demmings et al., 2021). A diagram of the unfolded protein response (UPR) pathways in ERS is shown in Figure 6A. In SCA5 and SCA14, pathogenic mutations promoted misfolding and aggregation of the disease-causing proteins (Armbrust et al., 2014; Takahashi et al., 2015; Avery et al., 2017), and even misfolded proteins may form inclusion bodies in some SCAs (Seidel et al., 2012). Similar aggregation phenomena can be found in patient-derived NPCs (Supplementary Figure 1). The result showed that the mutation caused the misfolding of the Kv4.3 and further increased protein degradation. This is consistent with previous reports (Duarri et al., 2012). At the same time, misfolded proteins activate the ERS pathway. Previous research has reported the intrinsic autonomous firing of cerebellar Purkinje neurons, and the neuronal inputs to Purkinje neurons were regulated by various ligand-gated and voltage-dependent ion channels, while the corresponding channel modulators alleviated the movement disorder (Hourez et al., 2011; Bushart et al., 2018).

The ERS is also involved in the pathogenesis of many other neurodegenerative diseases. The increase of many ERS markers can be detected in chronic traumatic encephalopathies (CTE) samples, such as CHOP and ATF6. The cognitive ability of the CTE rat model can be improved by using ERS inhibitor (Lucke-Wold et al., 2016). It was found that PERK activity increased in both Alzheimer's disease patients and mouse brain tissues (Abisambra et al., 2013). These findings provide a reference for the treatment of SCA19/22.

The c.1130 C>T (p.T377M) mutation in *KCND3* leads to neurotransmitter release and neuronal excitability dysfunction in neurons by affecting the function of potassium channels. Moreover, misfolded Kv4.3 mediates proteotoxicity and further activates the ERS pathway to result in neuronal apoptosis.

## Conclusion

The c.1130 C>T (p.T377M) loci mutation of *KCND3* mediated misfolding and the aggregation of Kv4.3, which activated the PERK-ATF4-CHOP pathway and further induced neuron apoptosis.

## Data Availability Statement

The data presented in the study are deposited in the China National GeneBank DataBase, repository, accession number CNP0003030.

## Ethics Statement

The studies involving human participants were reviewed and approved by First Affiliated Hospital of Zhengzhou University. The patients/participants provided their written informed consent to participate in this study. The animal study was reviewed and approved by First Affiliated Hospital of Zhengzhou University.

## Author Contributions

CS and YX contributed to the conception and design of the study and contributed to drafting the text and preparing the figures. CS, YX, FL, XH, JL, ZH, LF, SZ, and ML contributed to the acquisition and analysis of data. All authors contributed to manuscript revision, and read and approved the submitted version.

## Funding

This study was supported by the National Natural Science Foundation of China to CS (Grant Nos. 81974211, 82171247).

## Conflict of Interest

The authors declare that the research was conducted in the absence of any commercial or financial relationships that could be construed as a potential conflict of interest.

## Publisher's Note

All claims expressed in this article are solely those of the authors and do not necessarily represent those of their affiliated organizations, or those of the publisher, the editors and the reviewers. Any product that may be evaluated in this article, or claim that may be made by its manufacturer, is not guaranteed or endorsed by the publisher.
