# Supplementary material for: Rare KCND3 Loss-of-Function Mutation Associated With the SCA19/22
Source: Front Mol Neurosci. 2022 Jun 23;15:919199. doi: 10.3389/fnmol.2022.919199 (PMC9261871; doi:10.3389/fnmol.2022.919199)
Supplement: Supplementary file 2 [file Table_2.docx]

| Age of Onset | 28 (1–90) |
| --- | --- |
| First symptom | Ataxia (49)  Neurodevelopmental disorders/cognitive impairment (5)  Epilepsy (5)  Episodic ataxia (2)  Head tremor (2)  Intermittent diplopia (1)  Psychiatric symptoms (1) |
| Ataxia | 71/76 |
| Episodic ataxia | 3/76 |
| Neurodevelopmental disorders/Cognitive impairment | 36/76 |
| Movement disorder  Parkinsonism  Tremor  Myoclonus  Dystonia | 29/76  12  7  5  5 |
| Epilepsy | 7/76 |
| Pyramidal signs | 18/76 |
| Peripheral Neuropathy | 5/76 |

Supplementary Table 2. Clinical data of 76 patients KCND3 mutation carriers.
