# Supplementary material for: Rare KCND3 Loss-of-Function Mutation Associated With the SCA19/22
Source: Front Mol Neurosci. 2022 Jun 23;15:919199. doi: 10.3389/fnmol.2022.919199 (PMC9261871; doi:10.3389/fnmol.2022.919199)
Supplement: Supplementary file 3 [file Image_1.pdf]

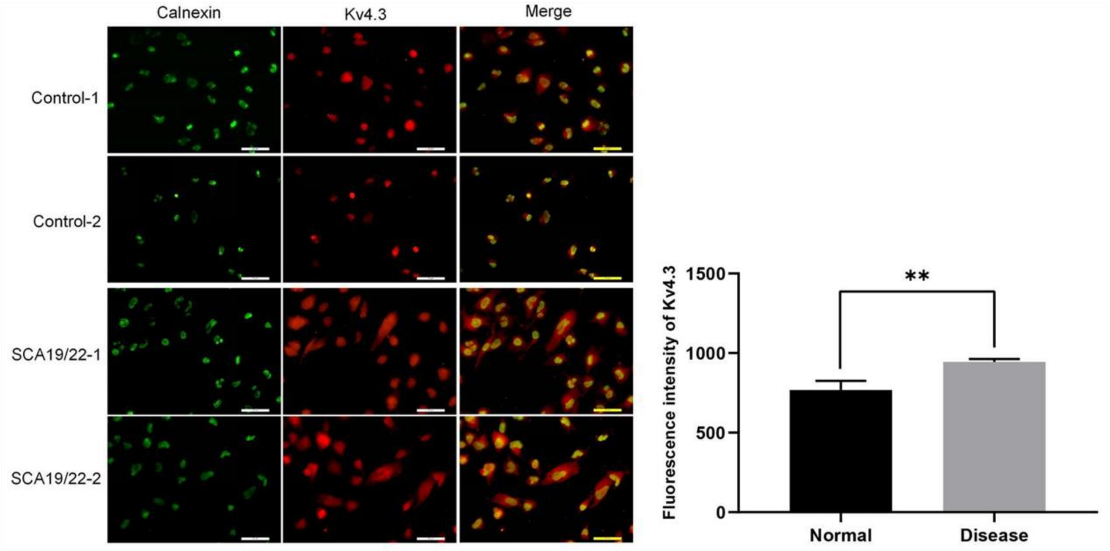

Supplement Fig. 1: Left: Colocalization with the ER marker calnexin (in green) revealed that mutant Kv4.3 proteins (in red) were retained in the ER, as was shown by the merged picture (in yellow). Scale bars 50um. Right: The results of fluorescence intensity analysis showed that more Kv4.3 was trapped in the NPCs in the disease group.
